# Genome-Wide Identification, Evolutionary and Functional Analyses of WRKY Family Members in *Ginkgo biloba*

**DOI:** 10.3390/genes14020343

**Published:** 2023-01-28

**Authors:** Weixing Li, Nan Xiao, Yawen Wang, Ximeng Liu, Zhaoyu Chen, Xiaoyin Gu, Yadi Chen

**Affiliations:** 1 College of Horticulture and Landscape, Yangzhou University, Yangzhou 225009, China; 2 Jiangsu Provincial Engineering Research Center of Green Horticulture, Yangzhou University, Yangzhou 225009, China

**Keywords:** *Ginkgo biloba*, WRKY, genome−wide identification, phylogenetic analysis, expression analysis, abiotic stresses

## Abstract

WRKY transcription factors (TFs) are one of the largest families in plants which play essential roles in plant growth and stress response. *Ginkgo biloba* is a living fossil that has remained essentially unchanged for more than 200 million years, and now has become widespread worldwide due to the medicinal active ingredients in its leaves. Here, 37 WRKY genes were identified, which were distributed randomly in nine chromosomes of *G. biloba*. Results of the phylogenetic analysis indicated that the GbWRKY could be divided into three groups. Furthermore, the expression patterns of *GbWRKY* genes were analyzed. Gene expression profiling and qRT−PCR revealed that different members of *GbWRKY* have different spatiotemporal expression patterns in different abiotic stresses. Most of the *GbWRKY* genes can respond to UV-B radiation, drought, high temperature and salt treatment. Meanwhile, all GbWRKY members performed phylogenetic tree analyses with the WRKY proteins of other species which were known to be associated with abiotic stress. The result suggested that GbWRKY may play a crucial role in regulating multiple stress tolerances. Additionally, GbWRKY13 and GbWRKY37 were all located in the nucleus, while GbWRKY15 was located in the nucleus and cytomembrane.

## 1. Introduction

The family of WRKY is a unique superfamily transcription factor (TF) of higher plants and algae, which plays vital roles in many life processes, especially in the protection of biological and abiotic stress [1,2]. WRKY structure consists of two parts, the N-terminal DNA binding domain and the C-terminal zinc-finger structure [3]. As a typical transcription factor, WRKY responds to various cues by recognizing the W box (C/T) TGAC (C/T) or W box−like (TGAC [C/T]) *cis*−regulatory elements in the promoters of the target genes to promote or inhibit genes expression. The WRKY TF superfamily are classified by their possession of the WRKY domain. The WRKY domain is consistent with the highly conserved heptapeptide WRKYGQK, known as the WRKY motif. Some plants have variants of the WRKYGQK motif, including WRKYSEK and WRKYGEK. The zinc-finger structure mainly includes C2H2 and C2HC type [4], but some of them exist in the form of CX29HXH and CX7CX24HXC [5]. According to the number of WRKY domains and the structure of their zinc-finger motifs, WRKY can be divided into I~III groups [4]. Group I WRKY TFs contains two WRKY domains and the zinc-finger structure is C2H2. Group II contains two WRKY domains, and the zinc-finger structure is C2H2 which is further divided into IIa, IIb, IIc, IId, IIe. Group III contains two WRKY domain, and the zinc-finger structure is C2HC [3,4,6]. Among them, the Group III WRKY TFs are considered the most evolutionarily advanced and adaptable [7].

In 1994, *SPF1* gene of WRKY family was first found in *Impoea batatas* [8]. With the development of genome sequencing technology, large numbers of WRKY have been identified in many plant species [5,6,9,10,11,12,13,14,15,16,17,18]. Plants often encounter adverse conditions, such as drought, critical temperature, and changes in light conditions. To adapt to these environments, plants have evolved complex mechanisms at many levels. At the molecular level, a large part of the genome is used for transcription. TFs are composed of a gene family of plant regulatory proteins with a wide range of expressions [19]. The WRKY protein is believed to regulate plant responses to abiotic stresses including cold, drought, heat, heavy metal toxicity, salt stress and UV-B radiation [20]. For example, AtWRKY25, AtWRKY26 could improve the tolerance of *Arabidopsis thaliana* to high temperature stress [20,21]. GmWRKY54 of *Soybean* could inhibit the expression of *STZ* and regulated the response to salt stress through *DREB2A*−mediated pathway [22]. OsWRKY76 of rice showed negative regulatory effects under low temperature stress [23]. In addition, the physiological response regulated by WRKY also includes some signaling substances such as ethylene (ETH), abscisic acid (ABA) and gibberellin (GA) [24]. Under drought stress, higher ABA levels were accumulated in plants and the stomata of leaves closed. AtWRKY63 of *A. thaliana* had a certain influence on ABA-mediated stomatal closure, thus affecting the drought response pathway [25]. The expression of *AtWRKY25*, *AtWRKY26* and *AtWRKY33* in *A. thaliana* could be induced by ETH, which regulates the expression of *EIN2* under high temperature. Furthermore, it can effectively activate the ETH-mediated signal transduction pathway and make plants have relatively stronger heat tolerance [21]. *G. biloba* is native to China and is regarded as a living fossil. *G. biloba* leaves are rich in terpenoids, flavonoids and proanthocyanidins and have been used for herbal medicine in China for thousands of years [26,27]. After the quaternary glacial catastrophe, Ginkgo has been widely planted all over the world, showing strong environmental adaptability and tolerance to extreme conditions [28,29]. Liao et al. [30] identified 28 WRKY genes from the transcriptome data of *G. biloba* based on the conserved WRKY domain and zinc-finger structure for the first time. It pointed out that *GbWRKY2* was preferentially expressed in flowers, and methyl jasmonate (MeJA) had a strong induction effect on it. GbWRKY2 and GbWRKY11 have been identified for sequence analysis, which revealed maximum gene expression in ovules and pollen cones, respectively. Subsequently, Liao [31] cloned and identified *GbWRKY11*, which is mainly expressed in female flowers and was induced by SA, ETH, ABA and other plant hormones, while the expression decreased under MeJA, salinity, low and high temperature treatment. GbWRKY20 encoded a newly cloned WRKY which responds to salt, drought, heat and plant hormones [32]. Guan [33] completed the whole genome sequencing of *G. biloba* in 2016. On this basis, 40 members of the WRKY family of *G. biloba* were identified in the latest research, and their expression patterns were explored by hormone and stress treatment [34]. In addition, some studies have shown that there are binding sites of WRKY protein in the promoter region of the ginkgolide biosynthesis gene, such as *GbLPS* [35], *GbDXS* [36], *GbGGPPS* [36], *GbHMGR* [37], *GbIDS* [38], *GbMECP* and *GbMECT* [39]. These studies provide a theoretical basis for the study of the anti-reversal function of Ginkgo WRKY family genes. However, due to the large Ginkgo genome and updated chromosome level in 2019, there are still many resistance genes not identified and the regulation of resistance is still unclear.

In the cultivation of *G. biloba*, abiotic stresses such as extreme temperature, salt and drought are the main limiting factors, which greatly affect the economic and ecological application [40]. Therefore, it is important to study the regulatory pathway and mechanisms of WRKY under abiotic stress. Through these studies, we can provide important gene resources for the cultivation of new stress resistant crop germplasm, which is conducive to the long-term and stable development of agriculture. In this study, to explore valuable evolutionary and functional information on the GbWRKY gene family in *G. biloba*, we identified 37 members of GbWRKY TF family of *G. biloba*, analyzed the expression patterns of GbWRKYs under five common abiotic stresses (high temperature, low temperature, drought, high salt, UV−B radiation), which provide references for the further study of the regulatory network of GbWRKY in abiotic stress response.

## 2. Materials and Methods

### 2.1. Plant Materials and Treatments

The seeds of G. biloba used in this study were collected from Yangzhou University (32° 20′ N, 119° 30′ E). G. biloba seedlings were cultured in the growth chamber at 25 °C with 16 h light. When the seedlings developed 5–6 leaves after 2 months, the seedlings were treated by high temperature (40 °C), low temperature (4 °C), salt stress (100 mmol/L), UV−B radiation (35 μw/cm^2^) and drought stress. Seedlings treated with distilled water were used as the control. Leave tissues were harvested from each sample with three biological replicates. The harvested leaves were immediately frozen in liquid nitrogen and stored at −80 °C for subsequent analysis.

### 2.2. Identification of WRKY Family Members in G. biloba

The Hidden Markov Model (HMM) [41] and a Simple Modular Architecture Research Tool (SMART) [42] were used in this study. The HMM profile was downloaded from Pfam protein family database. The domain contained in GbWRKY protein sequence was detected using the Hmmsearch program in HMMER software [43]. The results of the HMMER were screened to remove protein sequences that were 45% longer than the length of the HMM model domain, while retaining the longest protein sequence in the variable shear. All non-redundant protein sequences were retrieved and further analyzed with SMART (http://smart.embl-heidelberg.de/, accessed on 5 November 2021). Finally, 37 protein sequences corresponding to WRKYfamilies with conserved structural domains were obtained and named based on their position on the chromosome.

The Secondary structures of WRKY domains were performed by the website (https://www.novopro.cn/tools/secondary-structure-prediction.html, accessed on 6 November 2021).

### 2.3. Chromosome Localization of GbWRKYs

The chromosomal localization information was extracted from the *G. biloba* genome database and visualized using TBtools [44].

### 2.4. Prediction of Physicochemical Properties and Subcellular Localization

The physicochemical properties such as numbers of amino acids, MW (molecular weights), pI (isoelectric points), GRAVY (grand average of hydropathicity) of GbWRKY proteins were computed using the ProtParam software [45] (https://web.expasy.org/protparam/, accessed on 5 December 2021). The theoretical protein subcellular localization was conducted by Plant-mPLoc [46] (http://www.csbio.sjtu.edu.cn/bioinf/plant-multi/, accessed on 8 December 2021) and ProtComp [47] (http://www.softberry.com/berry.phtml?topic=protcomppl&group=programs&subgroup=proloc, accessed on 15 December 2021).

### 2.5. Gene Duplication

The potential replication genes were identified using the replicate gene classifier program of MCScanX software [48]. All the coding gene protein sequences of the genome were aligned using blastp, and the alignment results were used as input files for MCScanX software for replication gene prediction. The gene was identified as a replication gene according to e-value < 1 × 10^−5^ or e-value < 1 × 10^−10^. Afterwards, the genes were divided into segmental replication, tandem replication, proximal replication, and decentralized replication.

### 2.6. Nonsynonymous and Synonymous Substitution Rate Ratio (Ka/Ks)

TBtools software were used to analysis the Ks (synonymous substitution) and Ka (non-synonymous substitution). The differentiation time of GbWRKY gene replication was estimated by referring to Shen and Yuan’s paper [49].

### 2.7. Protein Conserved Domains and Motif Analysis

Pfam were used to analyze the conserved structural domains and position information of WRKY proteins [44], and the protein’s conserved motifs were performed by the online tool MEME (http://meme.nbcr.net/meme/, accessed on 20 December 2021). Tbtools were used for presenting the results.

### 2.8. Closely Related Species Analysis

The protein files of Ginkgo, Arabidopsis, rice, Selaginella and Picea were downloaded from PlantTFDB (http://planttfdb.gao-lab.org/, accessed on 24 December 2021). On this basis, the method for identification of GbWRKY proteins was used to search for WRKY members of other species. A total of 162 WRKY proteins were identified, including 60 members of Arabidopsis, 37 members in Ginkgo, 39 members in rice, 24 members in Selaginella, and 50 members in Picea. These protein sequences were used to construct phylogenetic trees and multiple sequence alignment [50]. Phylogenetic tree construction was based on ML (maximum likelihood) method [51] and via the IQ-TREE software [52,53] with 1000 bootstrap replicates.

### 2.9. Prediction of Promoter Cis-Acting Elements

The promoter sequences of target genes were extracted from the *G. biloba* genome, and *cis*-acting elements were predicted through the PlantCARE online tools.

### 2.10. RNA Extraction and Quantitative RT-PCR Analysis

Total RNA was extracted from *G. biloba* tissues after different stress treatments. RNA samples were processed according to the manufacturer’s ((Perfect Real Time, Takara, Japan) instructions. T Specific primers for RT-PCR were listed in Table S2, *GbGAPDH* were used as reference genes [54,55]. The 2^−ΔΔCt^ method was used for expression calculations [56].

## 3. Results

### 3.1. Identification of GbWRKY Transcription Factor Members in G. biloba

The WRKY family genes in *G. biloba* genome were identified by the HMMER3.0 software [43]. In total, 37 candidate WRKY genes were detected based on the chromosomal locations, the candidate GbWRKY genes were provisionally named GbWRKY1 to GbWRKY37 (Table 1).

The physiological and chemical characteristics of the GbWRKY members were investigated, including CDS (Coding Sequence), the protein molecular weight (MW) and isoelectric point (pI). The gene transcripts of the candidate *GbWRKY* genes range in size from 396 bp (*GbWRKY23*) to 3123 bp (*GbWRKY36*), encoding 131 to 1041 amino acids, respectively. pI distributions of GbWRKY proteins ranged from 4.72 (GbWRKY19) to 9.66 (GbWRKY14), indicating that the GbWRKY family members might play a role in different cellular microenvironments. The subcellular localization prediction results showed that most GbWRKY family members were nuclear localization proteins, and while one member existed in the extracellular space (GbWRKY24). The grand average of the hydropathicity (GRAVY) data of GbWRKY genes were negative, implicating hydrophilicity.

### 3.2. Chromosome Distribution and Gene Duplication Analysis of GbWRKY Genes

**Figure 1:**
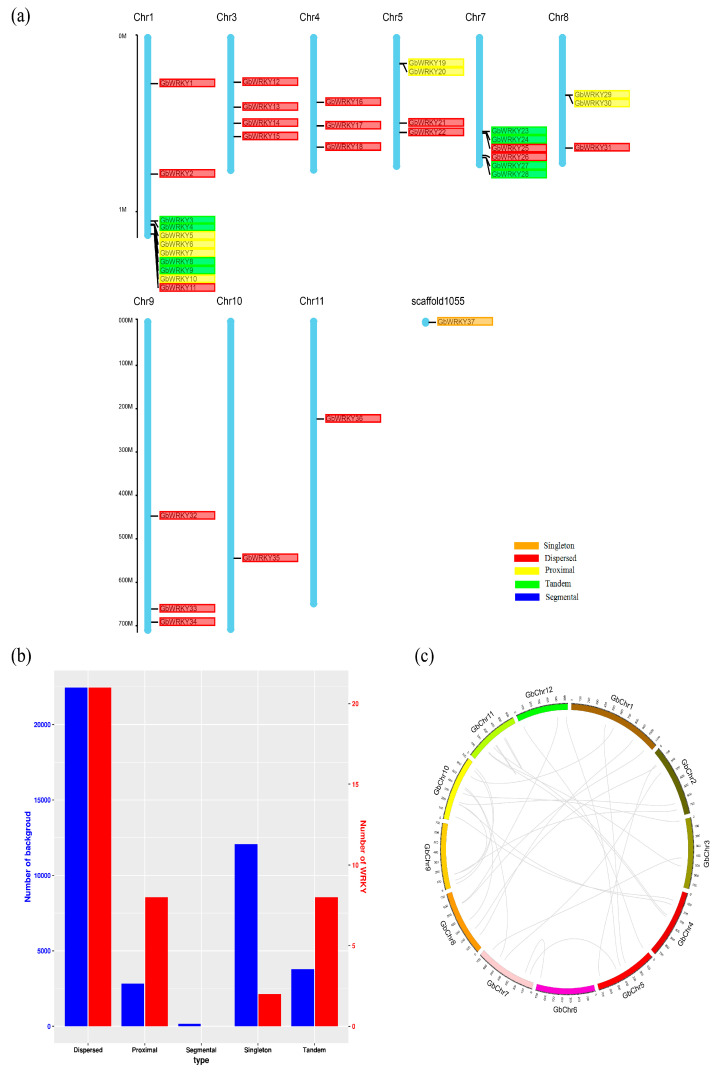
Chromosomal distribution and gene duplication of GbWRKYs genes. (**a**) Chromosomal distribution and gene duplication analysis of GbWRKYs, they were performed by Circos software and MCScanX, respectively; (**b**) statistical map of the number of replicating genes of each type; (**c**) collinearity analysis. The proximal duplicated genes are indicated in yellow. Tandem duplications are indicated in green.

The chromosomal location analyses, using Circos software [57], revealed that 37 candidate GbWRKY genes were unevenly distributed across nine chromosomes (1, 3, 4, 5, 7, 8, 9, 10 and 11Chr.), while GbWRKY37 was located on scaffold1055 (Figure 1a). The largest number of GbWRKY genes was observed in Chr. 1 (11), followed by Chr. 7 (6), Chr. 3 (4) and Chr. 5 (4), Chr. 4 (3), Chr. 8 (3) and Chr. 9 (3). On the contrary, Chr. 10 and Chr. 11 contained only one GbWRKY gene.

Gene duplication is one of the most important evolutionary processes producing new genetic material, and therefore new biological functions. Additionally, gene duplication promotes adaptation and speciation [58]. Underwent a genome duplication event, we found that among the 37 GbWRKY genes, GbWRKY3/GbWRKY4 on Chr.1 (location: 1053.17 Mb/1053.57 Mb), GbWRKY8/GbWRKY9 on Chr.1 (location: 1075.92 Mb/1077.67 Mb), GbWRKY23/GbWRKY24 on Chr.7 (location: 537.10 Mb/537.46 Mb), and GbWRKY27/GbWRKY28 on Chr.7 (location: 687.80 Mb/688.43 Mb) were tandem duplications (Figure 1a,b), by the definition of tandem replication [59]. Furthermore, 20 of 37 GbWRKY genes were dispersed, there are eight genes that were proximal copies, and one singleton gene (Figure 1a,b). In addition, the results of collinearity analysis of *G. biloba* showed that there were no collinearity pairs in the GbWRKY genes (Figure 1c).

The ratio of Ka (non-synonymous substitutions per non-synonymous site) to Ks (synonymous substitutions per synonymous site) is a valid indicator for examining positive selection pressure after gene replication is an effective indicator to test the positive selection pressure after gene duplication, meanwhile it provides inference for potential date of duplication events [60]. The GbWRKY3/GbWRKY4, GbWRKY8/GbWRKY9, GbWRKY23/GbWRKY24, GbWRKY27/GbWRKY28 Ka/Ks ratio were less than one (Table 2), suggesting that these four pairs of duplicated genes have undergone purification and elimination during evolution. The earliest divergence time between GbWRKY3 and GbWRKY4, GbWRKY8 and GbWRKY9 was around 0.47 million years ago (Mya), while GbWRKY23/GbWRKY24, GbWRKY27/GbWRKY28 began to diverge around 0.2 million years ago.

### 3.3. Structure and Sequence Alignment and Classification of GbWRKY Protein

**Figure 2:**
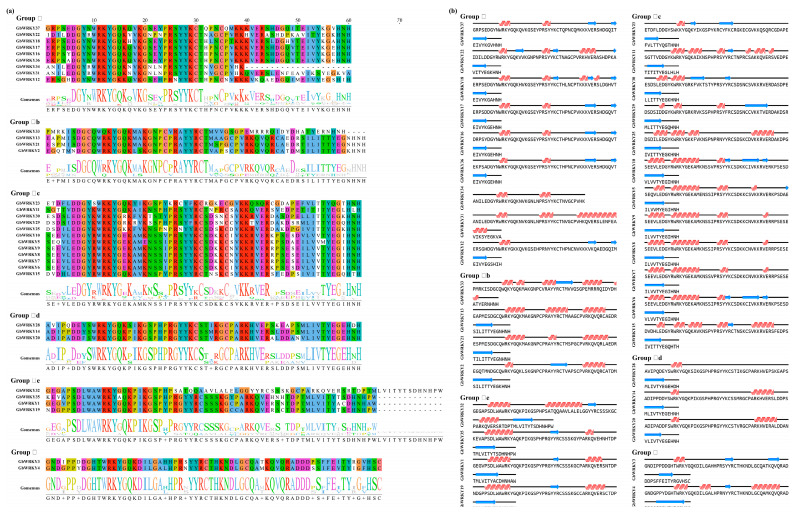
Multisequence alignment (**a**) and secondary structure (**b**) analysis of W−box in the GbWRKY proteins. (**a**) Identical amino acids are shown in same colors. (**b**) The secondary structures were conducted by NovoPro Bioscience Inc, an online tool.

According to the number of WRKY conserved domains and the type of zinc-finger structure, the WRKY proteins in *A. thaliana* were used to determine the grouping situation. Compared with *Arabidopsis* type Ⅰ clan, II clan, and III clan, the GbWRKY proteins are divided into three types: Ⅰ~III. The II type can be further divided into four subgroups based on the differences of WRKY conserved domain: IIb ~IIe. Group I include nine genes, these genes have zinc-finger structure types of C-X4-5-C-X22-23-H-X1-H (C2H2). Most of the GbWRKY genes belong to the II group, including 29 genes. Among the II group, each subgroup (IIb, IId, and IIe) contains four genes, while IIc includes 14 members, representing the largest subgroup (Figure 2a).

The structure type of the zinc finger of IId and IIe is C-X5-C-X23-H-X-H, IIb is C-X6-C-X29-H-X-H, and IIc is C-X4-C-X23-H-X-H. Group III has the lowest number of members and two genes in total, including GbWRKY3 and GbWRKY4. The structure type of the zinc finger is C-X4-C-X23-H-X-H. The protein sequence of GbWRKY34 was missing, but combined with the previous genome sequence, we finally classified it as a member of I group according to its characteristics. In addition, GbWRKY31, GbWRKY24, GbWRKY26 and GbWRKY27 have deletion or redundancy of protein sequences (Figure 2b). According to the evolutionary relationship of phylogenetic tree (Figure S1), it is speculated that the groups are I group, IIc group and IId group, respectively.

Sequence alignment software DNAMAN was used to compare the WRKY conserved domain of 37 GbWRKY proteins. GbWRKY12 in IIc group and GbWRKY25 in I group contain WRKYGKK structure, GbWRKY33 of IIb group contain WQKYGQK structure, GbWRKY30 and GbWRKY29 of IIc group contain WRKYGRK structure, GbWRKY10, GbWRKY5, GbWRKY9, GbWRKY8, GbWRKY7 and GbWRKY6 contain WRKYGEK structure, and GbWRKY35 of IIe group contain WRKYAQK structure (Figure 2). These may have mutated during evolution.

### 3.4. Phylogenetic Analysis, Conserved Motifs and Exon−Intron Organization of GbWRKY Family Members

The phylogenetic analysis of the 37 GbWRKY protein sequences was carried out by IQ-TREE [52,53] to further investigate the evolution relationship of GbWRKY members (Figure S2a). Furthermore, we analyzed the number and length of the GbWRKY gene introns and exons. The number of introns ranged from 1 to 19; in addition, the size of introns showed some extents of difference, as shown in Figure S2b. Compared with *GbWRKY34*, *GbWRKY23* and *GbWRKY7*, *GbWRKY36* and *GbWRKY32* had longer introns.

Although most closely related genes showed high similarity and conservation in structures, there are still some differences in the numbers and length of introns among some phylogenetic ally related members. *GbWRKY17* and *GbWRKY16* were related genes according to the polygenetic analysis, they had three or two short introns, while *GbWRKY36* had 16 longer introns, which may cause the expression pattern and function of *GbWRKY36* to be different from that of *GbWRKY17* and *GbWRKY16*. Moreover, from the distribution position of exons in nucleic acid sequence, the exon length of genes with more exons is generally shorter, and the distribution of exons in full-length sequence is more dispersed than that of other *GbWRKY* family members. On the other hand, the exon length of genes with fewer exons is generally longer, and the distribution of exons on the full-length sequence is more compact. In addition, none of the 37 *GbWRKY* family members had UTR structure. The GbWRKY proteins contain 15 conserved components, and 36 members all have the typical heptapeptide conserved domain (Figure S2b). Most of the members included Motif1 except for GbWRKY16, GbWRKY33, and GbWRKY34, all members contain Motif2. The motif sequences are listed in Table S1.

### 3.5. Conserved Domain Evolution of G. biloba and Multispecies WRKY Proteins

**Figure 3:**
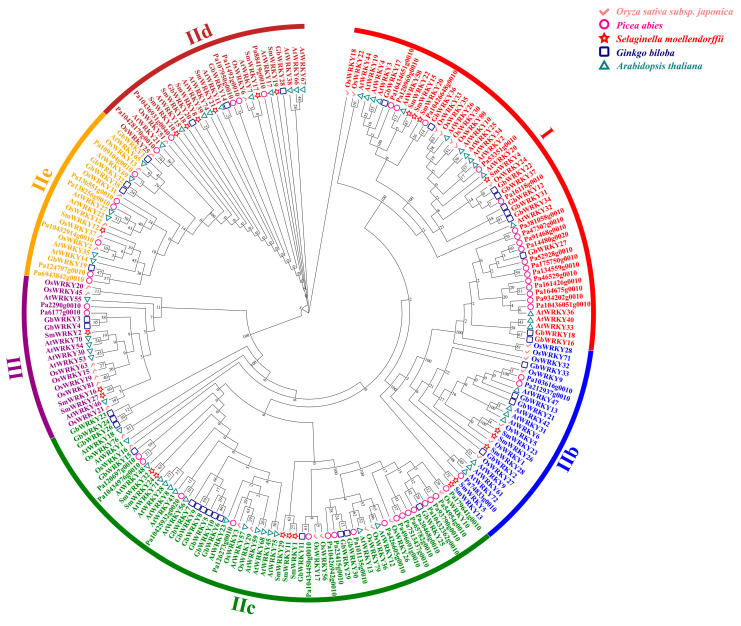
The phylogenetic tree of the WRKY proteins in *G. biloba*, *A. thaliana*, *O. sativa*, *S. moellendorffii* and P.abies.3.6. Tissue−specific expression analysis of GbWRKY genes.

*G. biloba* has a special evolutionary relationship, which is a single family, a single genus, and a single species [61]. Then, we investigated the duplication and diversification of GbWRKY gene family. A phylogenetic tree of WRKY complete protein sequences from five representative species, including one monocot (*Oryza sativa*), one eudicot (*A. thaliana*) and three ferns (*Selaginella moellendorffii*, *gymnosperm*, and *Picea abies*), was constructed using MEGA 5.0 (Figure 3). In general, the number of GbWRKY genes is similar to the *P. abies*, and significantly lower than *A. thaliana* and *O. sativa*, while the *S. moellendorffii* WRKY gene are significantly lower than that of gymnosperms and angiosperms. The phylogenetic analysis suggested that the number of WRKY genes is between species. For example, in Group III, the *G. biloba* WRKY gene family contains two members: *GbWRKY3* and *GbWRKY4*, and the *S. moellendorffii* and *P. abies* also contain two members, while the *Arabidopsis* and *O. sativa* contain 14 and 12 family members, respectively. There are also many branches of evolution with high self-extension values that often contain the same numbers of WRKY genes from each species, suggesting that these WRKY genes may have existed before the species divergence. For example, OsWRKY5, OsWRKY1, AtWRKY31, AtWRKY42, AtWRKY6, and AtWRKY47. Some branches lack *GbWRKY* genes, such as the Group IIa branch, this supports that IIa genes are the most recent evolutionary generation [7]. This might be due to the loss of the corresponding WRKY member in *G. biloba* during the evolutionary process or caused by problems in the assembly or annotation of genomic data. In addition, WRKY genes with direct homologous relationships tend to be clustered together more than those with collateral homologous relationships, such as GbWRKY10, GbWRKY5, GbWRKY9, GbWRKY8, GbWRKY7, and GbWRKY6 genes in *G. biloba*.

**Figure 4:**
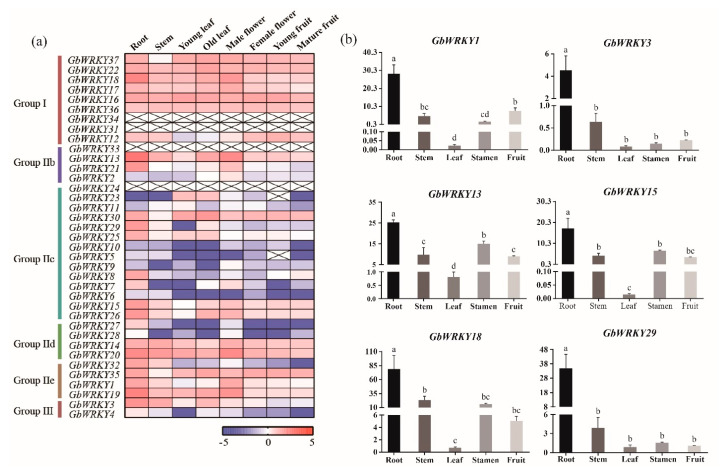
Expression profiles of *GbWRKY* genes in different *G. biloba* tissues from RNA−Seq and DGE data (**a**) and qRT−PCR verification (**b**).

The expression pattern of *GbWRKY* genes in different tissues showed that the expression levels of individual members of this gene family varied significantly in various tissues (Figure 4). *GbWRKY16/17/18/22/37* in Group I were closely related WRKY genes on the phylogenetic tree, and they were highly expressed in all tissues under study. In Group IIc, the gene expression levels in all tissues were generally lower than that of others, such as *GbWRKY5*, *GbWRKY6*, *GbWRKY9* and *GbWRKY10*. In Group IId, *GbWRKY27* and *GbWRKY28* showed lower expression in multiple tissues, while *GbWRKY14* and *GbWRKY20* in the same group were highly expressed in all tissues. *GbWRKY20/37* showed in male or female flowers, implying that these genes may play an important role in flower formation and development. However, some of the members such as *GbWRKY13/14/16/17/18/19/20/22/36* were highly expressed in vegetative tissues, inferring that they may participate in the regulation of plant vegetative growth. Most of the GbWRKY genes were expressed highly in roots, such as *GbWRKY8*, which was expressed at higher rates in roots than in other organs, suggesting their possible involvement in *G. biloba* root development. On the contrary, *GbWRKY5*, *GbWRKY9*, and *GbWRKY10* in Group IIc, three closely related WRKY genes on the phylogenetic tree, have low expression in roots. In addition, some closely related *GbWRKYs* genes have different expression patterns, for example, *GbWRKY8* and *GbWRKY9* were predicted in tandem duplication but they differed in expression patterns. *GbWRKY8* was mainly expressed in roots, while *GbWRKY9* was highly expressed in male flowers (Figure 4a).

To validate the reliability of RNA−Seq and DGE data, qRT−PCR assays were randomly performed on six selected genes (Figure 4b). As expected, in our study, we found that the relative expressions of all the test genes are highly expression in roots. The expression trends of the selected genes are consistent with the presented data, indicating that the DGE and RNA−seq data were highly reliable.

### 3.6. Analysis of Cis−Acting Elements in the Promoter Region of GbWRKYs

**Figure 5:**
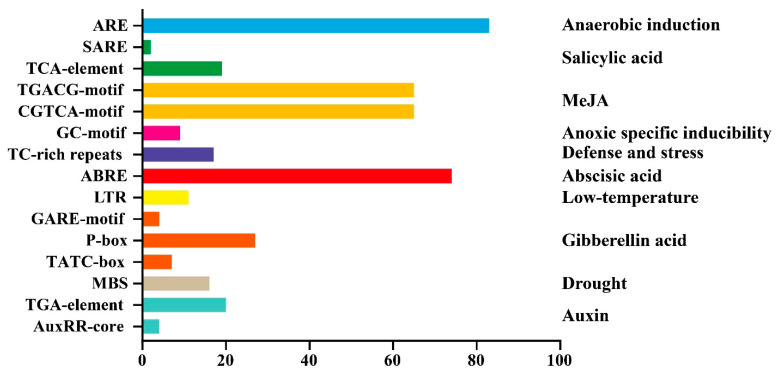
The number of *cis*−acting elements was tested in the promoter region of GbWRKYs. The name of *cis*−acting elements was listed on the left side of the image, and the corresponding function annotation was listed on the right side.

We analyzed the *cis*−acting elements in the 2000 bp sequence of the promoter region by using the PlantCARE online tool. Fifteen types *cis*−acting elements associated with responses to environmental stresses and plant hormones were identified in all *GbWRKY* promoters, such as defense and stress responsive element (TC-rich repeats), drought responsive element (MBS), and ABA response element (ABRE) (Figure 5). These results suggested that GbWRKYs may play a crucial role in plant growth and stress responses.

### 3.7. RNA−Seq Expression Analysis of GbWRKY Genes under Different Stress

**Figure 6:**
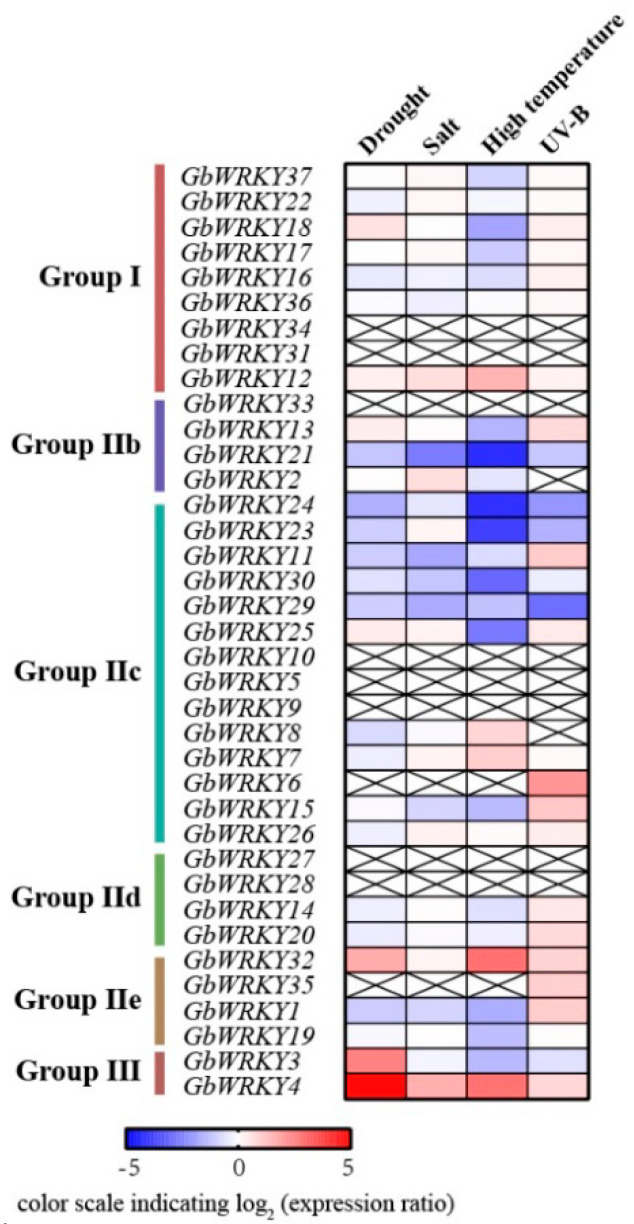
Analysis of expression patterns of *GbWRKY* genes under drought, salt, high temperature and UV−B radiation. The data were obtained from the publicly dataset. In the cDNA library of leaves under stress treatment, q-value < 0.05 and log2|fold−change| > 1 were used as the criteria to screen the differentially expressed WRKY genes.

To detect the expression of GbWRKY in different tissues, we performed RNA sequencing analysis using whole *G. biloba* with treatments [62,63]. As shown in Figure 6, most of the *GbWRKY* genes could respond to abiotic stress. The number of up-regulated *GbWRKYs* induced by salt and UV-B radiation was higher than that of down-regulated *GbWRKYs*. The *GbWRKY13/14/19/25/26* expression levels were induced under both salt stress and UV-B radiation, while *GbWRKY1/15/23* were down-regulated under both treatments compared with the control. In addition, *GbWRKY4/7/8/12/32* were dominantly GbWRKY genes during high temperature stress. Furthermore, closely related genes had similar expression patterns. For example, the *GbWRKY16* and *GbWRKY17* genes of Group I are closely related, and their expression patterns are similar under different abiotic stresses. They are down−regulated under drought and high temperature stress, and up-regulated under UV−B radiation, while some genes have different degree of response to different abiotic stresses. The expression level of *GbWRKY4* was increased under salt, high temperature and UV−B radiation, while *GbWRKY3* displayed decreased expression under these treatments compared with the control. The expression levels of *GbWRKY5/9/10/27/28/31/33/34* were very low in these treatments.

### 3.8. Expression Patterns of GbWRKY Genes Verified by qRT−PCR under Different Stresses

**Figure 7:**
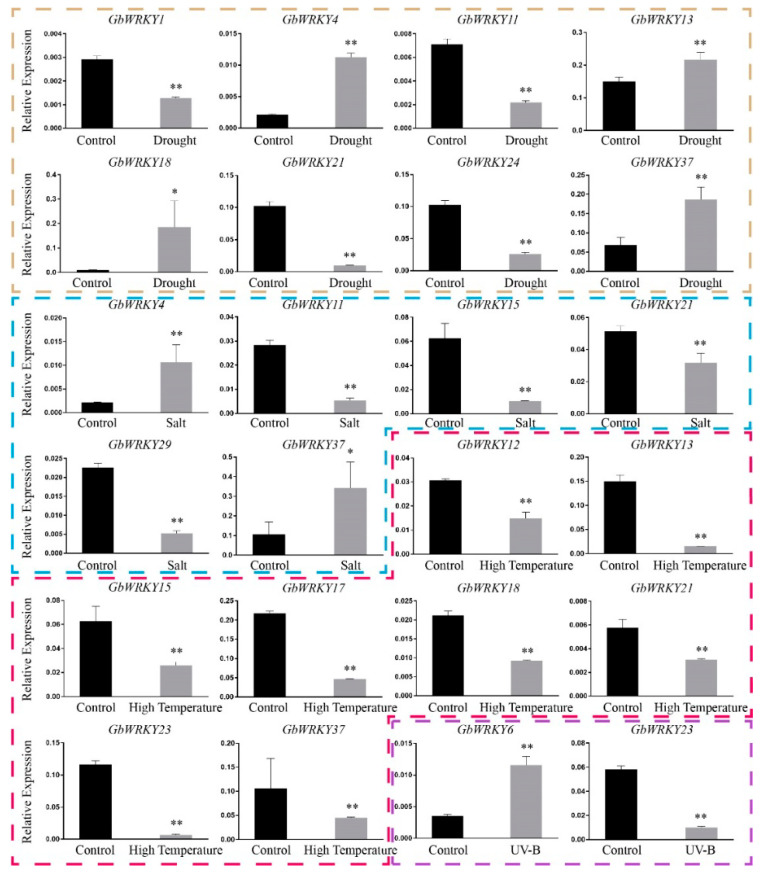
Expression patterns of selected GbWRKY genes under different stress by qRT−PCR, the data were normalized using the *GAPDH* gene. The error bars indicate the mean ± SD (*n* = 3). Asterisks indicate a significant difference as determined by a Student’s *t*-test (* *p* < 0.05, ** *p* < 0.01).

Based on the *cis*-acting elements analysis, the promoters of *GbWRKYs* contain a large number *cis*-acting elements which are related to stress. In our study, we conducted a variety of adverse treatments on *G. biloba* and analyzed the expression changes of *WRKY* genes. We found that *GbWRKY* genes were differentially expressed under different stress treatments. Under drought stress, the expressions of *GbWRKY4*, *GbWRKY13*, *GbWRKY18* and *GbWRKY37* were significantly up−regulation, and the expressions of *GbWRKY1*, *GbWRKY11*, *GbWRKY21* and *GbWRKY24* were significantly down-regulation. Under salt stress, *GbWRKY4* and *GbWRKY37* were significantly up-regulation, and *GbWRKY11*, *GbWRKY15*, *GbWRKY21* and *GbWRKY29* were significantly down-regulation. Under high temperature stress, *GbWRKY12*, *GbWRKY13*, *GbWRKY15*, *GbWRKY17*, *GbWRKY18*, *GbWRKY21*, *GbWRKY23*, and *GbWRKY37* were significantly down-regulation. Under UV−B radiation, *GbWRKY6* was significantly up-regulation and *GbWRKY23* was significantly down-regulation (Figure 7). The results of fluorescence quantification were consistent with the transcriptome data. Among the differentially expressed genes, *GbWRKY11*, *GbWRKY13*, *GbWRKY15*, *GbWRKY18*, *GbWRKY21*, and *GbWRKY37* could respond to multiple abiotic stresses.

### 3.9. Analysis of WRKY Gene Evolution Tree of G. biloba WRKY Family and Different Species with Known Abiotic Stress

**Figure 8:**
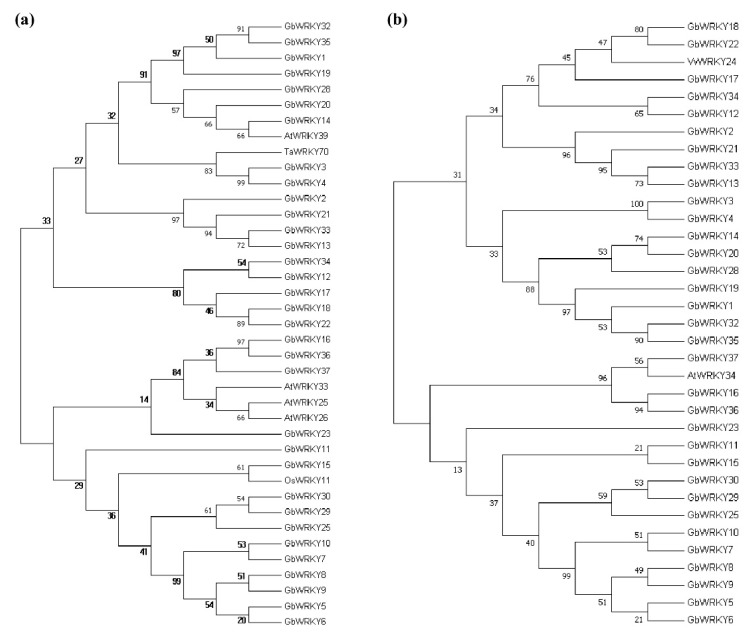
Phylogenetic tree of GbWRKYs and known WRKYs associated with high temperature stress (**a**) and low temperature stress (**b**).

**Figure 9:**
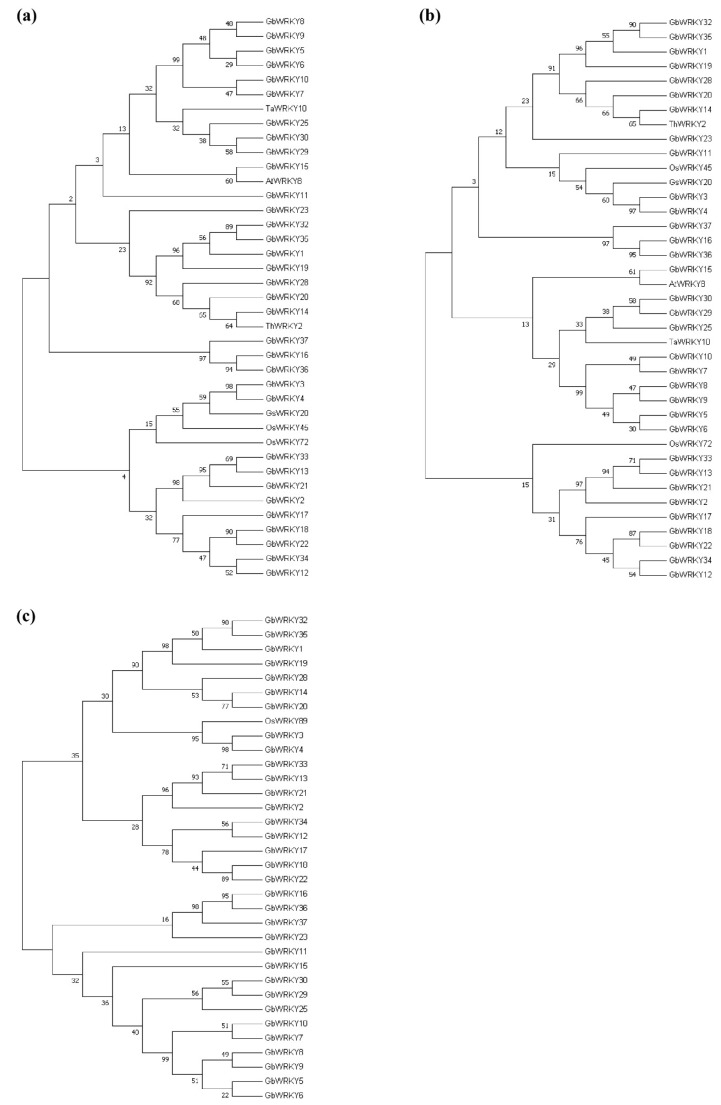
Phylogenetic tree analysis of GbWRKYs and known WRKYs related to salt (**a**), drought (**b**) and UV−B radiation (**c**).

To identify the stress-responsiveness GbWRKY genes, phylogenetic tree analysis was performed between 37 family members and WRKY genes related to known abiotic stress in other species. In extreme temperature stress, the related genes of phylogenetic tree analysis showed that the ginkgo GbWRKY15 with rice OsWRKY11, the ginkgo GbWRKY18 with grape VvWRKY24, and the ginkgo GbWRKY37 and Arabidopsis AtWRKY34 are close relatives (Figure 8), suggesting that these three genes in ginkgo might resist extreme temperature stress. The phylogenetic tree analysis of genes related to salt and drought stress showed that ginkgo GbWRKY15 was closely related to *Arabidopsis* AtWRKY8, and ginkgo GbWRKY13 was closely related to rice OsWRKY72, suggesting that these two genes played an important role in salt tolerance and drought resistance of *G. biloba* (Figure 9a,b). In addition, the GbWRKY family was far related to the rice OsWRKY89 gene associated with UV-B radiation (Figure 9c). The transcriptomic data and fluorescence quantitative results speculated that four genes of the GbWRKY family (GbWRKY13, GbWRKY15, GbWRKY18 and GbWRKY37) could be a response to abiotic stress such as extreme temperature, salt damage and drought, and GbWRKY6 and GbWRKY23 are involved in response to UV-B radiation.

### 3.10. Subcellular Localization of GbWRKY13/15/37

**Figure 10:**
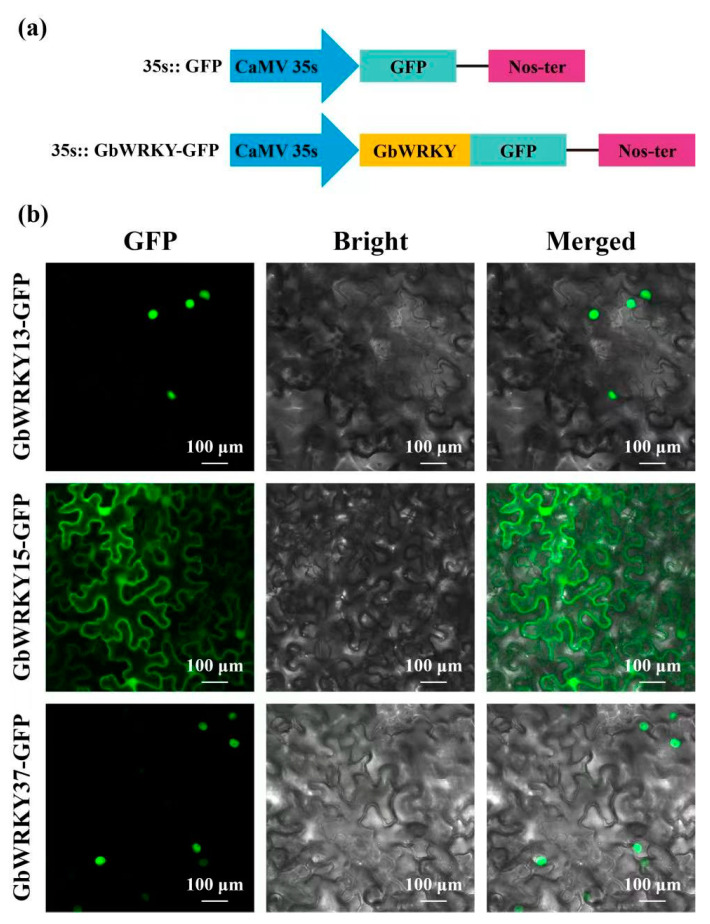
Subcellular localization of GbWRKY proteins. (**a**) Schematic illustration of the constructs. (**b**) Subcellular location of free GFP and GbWRKY-GFP protein in tobacco leaves.

Since subcellular localization information can provide some clues for protein function research, GbWRKY13, GbWRKY15, and GbWRKY37 may play important role in abiotic stress. We cloned the *GbWRKY13*, *GbWRKY15*, and *GbWRKY37* full−length CDS sequence. The sequence of *GbWRKY13* and *GbWRKY15* are consistent with the reference sequence, *GbWRKY37* expected length is 2586 bp, and the actual sequencing length is 1173 bp (Figure S3), it may be due to the duplication of micro-fragments that lead to the misassembled of the assembly. The full−length CDS without the termination codon was fused in−frame to the 5′ end of the GFP gene under the control of the CaMV 35s promoter (Figure 10a). The recombined vector pK2GW7-GbWRKY-GFP was transfected into tobacco leaves. Confocal microscopy revealed that GFP control displayed ubiquitous distribution in the whole cell, while the fusion protein GbWRKY13/37−GFP was detected specifically in the nucleus (Figure 10b). Furthermore, the results showed that GbWRKY15 protein was localized to the cytomembrane and nucleus.

## 4. Discussion

WRKY proteins have been proven to be important regulators in numerous processes of plants. Chromosomal localization analysis showed that, except for chromosomes 2, 6, and 12, GbWRKY distributed on the remaining 9 chromosomes (non-uniform distribution) (Figure 1). GbWRKY37 could not determine the chromosome, this phenomenon was also found in grapes [64]. In the long-term evolution, Ginkgo needs to face natural selections resulting in the deletion of the WRKY gene on chromosomes 2, 6, and 12; or it could be due to the accuracy of this genome sequencing. Tandem duplication of WRKY transcription factors is common in many plants, such as Arabidopsis, rice, poplar, grape [64,65,66]. In this study, there were eight genes that were in tandem duplication in *G. biloba* WRKY family members. It can provide some references for the in-depth study of the similarities and differences of the duplicated gene.

Previous research have uncovered the functions of WRKY genes in many plant species, such as Arabidopsis, tomato, rice, and pineapple. WRKY exist as gene families in plants, and the WRKY genes in different species were uneven. Herein, 37 members of GbWRKY genes were discovered at the chromosome level which is more than 28 candidate WRKY genes were identified in *G. biloba* in a previous study [30]. The number of GbWRKY in *G. biloba* is lower than that in other plants, such as *Arabidopsis* [67], *O. sativa* [10], *Solanum lycopersicum* [5], *Pinus monticola* [13], *Populus* [68], but is higher than that in *Platycodon grandiflorus* [69]. Phylogenetic analysis suggested that the GbWRKYs can be classified into three primary groups (I, II, and III) and five subgroups (IIa, IIb, IIc, IId, and IIe) based on the conserved WRKY gene domain along with the zinc-finger motif. There were 9 GbWRKYs in Group I, 26 in group II, and 2 in group III (Figure 2). The WRKY genes distribution among different plants was different. Grape has a large group II [70], and a similar WRKY distribution was found in Ginseng [71]. The group III has more members in *Arabidopsis*, maize and rice compared to that in other plants [72,73,74]. WRKY genes are thought to play an important role in plant evolution and adaptation [75]. Because of the fewer genes in group III compared with other plants, the GbWRKY expansion in *G. biloba* may be caused by the expansion of other WRKY groups.

Most WRKY proteins in *G. biloba* contain a highly conserved heptapeptide (WRKYGQK) at the N-terminus DNA binding domain, while variants WRKYGKK was found in *GbWRKY12* and *GbWRKY25*. Furthermore, *GbWRKY5*, *GbWRKY6*, *GbWRKY7*, *GbWRKY8*, *GbWRKY9*, and *GbWRKY10* which belong to the IIc group have the heptapeptide variant (WRKYGEK), consistent with the mutant form commonly found in rice. *GbWRKY29* and *GbWRKY30* belong to the IIc group and contain WRKYGRK variation, *GbWRKY33* contains WQKYGQK variation, and *GbWRKY35* contains WRKYAQK variation (Figure 2), which may be mutated during evolution and belong to new mutants. Variants were also found in *Arabidopsis*, apple, peach and other plants, indicating the diversity of plant WRKY gene family in the evolutionary process. The variation might affect the ability of WRKY genes to bind to the W-box. For example, the variants appeared in soybean and *Nicotiana tabacum* do not properly bind to the W-box [76,77]. All GbWRKY proteins contained more than two conserved motifs, consistent with studies from other plant species [78,79]. The members distributed in each group had the same or similar motif composition, suggesting that WRKY genes in the same group have similar protein structures and biological functions.

Plant WRKY proteins belong to the superfamily of zinc finger transcription factors and are plant specific [80]. Although the WRKY gene is regarded as a transcription factor unique to vegetation, it is also present in some non-plant species, such as *Chlamydomonas reinhardtii*, *Dictyostelium* and *Giardia lamblia* [6,81,82]. The WRKY genes contained in these three species and lower species such as mosses and gymnosperms cycads belong to family I, indicating that family I WRKY may be the ancestral class of the WRKY family [1]. Some studies suggest that the model WRKY gene containing a single WRKY structural region generates family I WRKY genes after domain cycling, followed by loss of the N-terminal domain to generate family IIc genes, and family III is the last evolution [58]. However, with the release of the genome sequences of *Moss* [12] and *Selaginella* [83], it was found that the genomes of *Selaginella japonicus* and moss all have Group III genes, while the genomes of *S. japonicus* are missing Group IIa genes, indicating that Group IIa genes are the most important. Late evolution produced [6] *G. biloba* was only left in China after the quaternary glacier catastrophe, and its evolution status is controversial because of its morphological characteristics different from other gymnosperms. Due to its special evolutionary status, this study compared the sequences of *G. biloba* with the gymnosperm *Norway spruce*, the fern *S. japonicus*, the monocotyledonous plant rice, and the dicotyledonous plant *A. thaliana*. The number of WRKY gene families is close, significantly lower than that of rice and *Arabidopsis* WRKY genes. The number of WRKY genes in *S. japonicus* was significantly lower than that in angiosperms and gymnosperms (Figure 3). It is inferred that the seed plants acquired new genes in the evolution process of lower plants, and at the same time, there was a loss and gain of individual genes in the evolution process of their respective species. Interestingly, similar to Jiangnan Selaginella [83], the *G. biloba* WRKY gene only lacks Group IIa genes, and our results support that Group IIa genes evolved last.

RNA−seq is an important approach for gene function to uncover new molecular aspects of particular processes. RNA−seq results indicated that *GbWRKYs* have different expression patterns in the eight tissues. These results suggested that GbWRKYs may perform a wide range of functions during the *G. biloba* lifecycle. Among 37 *GbWRKY* genes, 33 *GbWRKYs* were expressed in at least one tissue, and the *GbWRKYs* expression levels were diverse. In all the 37 *G. biloba* WRKY genes detected in this study, 17 genes, including *GbWRKY13*, *GbWRKY16*, *GbWRKY18*, and *GbWRKY20*, were highly expressed in roots, stems, leaves, flowers, and fruits. In addition, some genes displayed specific expression patterns. For instance, *GbWRKY27* and *GbWRKY28* displayed lower expression in multiple tissues, while *GbWRKY14* and *GbWRKY20* in the same group were expressed at a higher rate in almost all tissues. *GbWRKY20* and *GbWRKY37* showed in male or female flowers, other members such as *GbWRKY13/14/16/17/18/19/20/22/36* showed high expression levels in vegetative organs, such as roots, stems, and leaves. Most of the GbWRKY genes were highly expressed in roots, such as *GbWRKY8*. On the contrary, *GbWRKY5*, *GbWRKY9*, and *GbWRKY10* in Group IIc have low expression in roots (Figure 4), it is presumed that these *GbWRKY* genes may play crucial roles in these organs. These tissue-specific expression patterns suggest that GbWRKY is possibly involved in different tissue development.

During the long-term evolution, the plants have to deal with various environmental stimulates, such as salt, drought or UV−B radiation. Previous studies have suggested that WRKY genes play a crucial role in the response to abiotic stress [2,40]. For instance, 7 *GbWRKYs* responded to drought treatment, 5 GbWRKYs responded to salt treatment, 16 GbWRKYs changed under high temperature, and 7 *GbWRKYs* changed under UV-B radiation. Some genes such as *GbWRKY1*, *GbWRKY11*, *GbWRKY29* responded to all treatments. These WRKY may be the central TFs response to environmental stress (Figure 6). In tobacco, overexpression of *TaWRKY44* could enhance tobacco drought stress and salt stress [84]. GmWRKY21 and GmWRKY54 overexpression plants display enhanced salt, drought, and cold tolerance [22]. The GbWRKYs may play crucial roles in enhancing the tolerance of environmental stress. Furthermore, combined with transcriptome data, fluorescence quantitative results and phylogenetic tree analysis, it is speculated that four genes in the ginkgo WRKY family, GbWRKY4, GbWRKY12, and GbWRKY32, play important roles in responding to abiotic stresses. (Figure 6). In tobacco, overexpression of TaWRKY44 could enhance tobacco osmotic stress, salt stress and drought stress [84]. Overexpression of GmWRKY21 and GmWRKY54 shows enhanced salt, drought, and cold tolerance [22]. The GbWRKYs may play a crucial role in enhancing the tolerance of abiotic stress. Furthermore, combined with transcriptome data, fluorescence quantitative results and phylogenetic tree analysis, it is speculated that four genes in the ginkgo WRKY family, *GbWRKY4*, *GbWRKY12*, and *GbWRKY32*, play a key role in responding to abiotic stresses such as extreme temperature, salinity, and drought. *GbWRKY6* and *GbWRKY15* play a role in responding to UV-B radiation, and these genes can respond rapidly to stress treatment (Figure 8 and Figure 9). In addition, the functions of different types of WRKY transcription factors also have different emphases, and there are also differences in the way of regulating and controlling physiological metabolism [80]. Subcellular localization prediction and experimental results show that *GbWRKY13*, *GbWRKY15*, and *GbWRKY37* are localized in the nucleus and have strong fluorescence (Figure 10), indicating that these three genes mainly play a regulatory role in the nucleus, which is consistent with transcription factor properties.

## 5. Conclusions

In this study, we performed genome-wide identification of WRKY genes in G. biloba and their expression patterns in different tissues and responses to different stresses. A total of 37 WRKY family genes with three groups were identified in the genomes of *G. biloba.* Molecular evolutionary analysis showed that the WRKY gene structures were conserved in *G. biloba*. Gene expression profiling and qRT−PCR revealed that different groups of GbWRKYs have distinct spatiotemporal patterns of expression in normal conditions or when subjected to extreme temperature, salinity, and drought and UV−B radiation. Notably, we found a variety of *cis*−acting elements with diverse biological functions in the promoters of these genes, transcriptome data and qRT−PCR experiments confirmed that they have vital roles in hormone signaling to regulate the tolerance to abiotic and biotic stresses in *G. biloba*. The extensive annotation and expression analysis of the GbWRKYs contributes to our understanding of the functions of these genes in multiple stress responses and phytohormone crosstalk.

## Figures and Tables

**Table 1:** Genome−wide identification of GbWRKY genes.

Name	ID	Chromosomal Location	CDS (bp)	GRAVY	Subcellular Localization	MW (KDa)	pI
*GbWRKY1*	*Gb_05176*	261561178-261563292	1494	−0.814	Nucleus	54.67	6.53
*GbWRKY2*	*Gb_02351*	783125879-783138739	1581	−0.593	Nucleus	56.24	7.20
*GbWRKY3*	*Gb_00547*	1053172260-1053174875	1740	−0.538	Nucleus	63.67	6.58
*GbWRKY4*	*Gb_00545*	1053573972-1053576514	1446	−0.620	Nucleus	53.13	5.38
*GbWRKY5*	*Gb_40207*	1072194964-1072196293	801	−0.666	Nucleus	29.95	7.69
*GbWRKY6*	*Gb_26411*	1075330508-1075331449	747	−0.793	Nucleus	27.90	7.70
*GbWRKY7*	*Gb_26412*	1075829444-1075830197	573	−0.949	Nucleus	21.53	9.03
*GbWRKY8*	*Gb_26413*	1075925548-1075926489	747	−0.776	Nucleus	27.96	8.20
*GbWRKY9*	*Gb_40261*	1077671233-1077672112	681	−0.711	Nucleus	25.77	8.83
*GbWRKY10*	*Gb_40257*	1078258461-1078259371	723	−0.830	Nucleus	27.48	6.76
*GbWRKY11*	*Gb_17074*	1129012414-1129014214	1404	−0.739	Nucleus	51.72	4.89
*GbWRKY12*	*Gb_36273*	253690691-253784117	2621	−0.573	Nucleus	96.08	8.83
*GbWRKY13*	*Gb_39366*	397260590-397263274	2124	−0.651	Nucleus	76.52	6.11
*GbWRKY14*	*Gb_16513*	490181118-490183897	1083	−0.786	Nucleus	40.49	9.66
*GbWRKY15*	*Gb_01873*	567507034-567508969	1458	−0.721	Nucleus	52.55	5.73
*GbWRKY16*	*Gb_20926*	369244325-369246968	1443	−0.651	Nucleus	50.98	8.53
*GbWRKY17*	*Gb_31953*	505067926-505086750	1980	−0.866	Nucleus	71.11	6.86
*GbWRKY18*	*Gb_25118*	628096908-628105595	3046	−0.410	Nucleus	110.84	7.18
*GbWRKY19*	*Gb_25547*	145264191-145266777	1152	−0.717	Nucleus	42.17	4.72
*GbWRKY20*	*Gb_01527*	147519959-147521498	1104	−0.507	Nucleus	39.72	9.22
*GbWRKY21*	*Gb_23334*	489258288-489261466	2064	−0.745	Nucleus	74.81	5.92
*GbWRKY22*	*Gb_32055*	543409418-543471462	2328	−0.686	Nucleus	83.42	6.11
*GbWRKY23*	*Gb_17623*	537103484-537103996	396	−1.130	Nucleus	14.68	8.87
*GbWRKY24*	*Gb_15790*	537465845-537467456	534	−0.490	Extracellular	20.01	9.30
*GbWRKY25*	*Gb_16917*	546983507-546984721	822	−0.841	Nucleus	30.88	8.10
*GbWRKY26*	*Gb_08731*	675694495-675695882	1194	−0.598	Nucleus	43.05	5.60
*GbWRKY27*	*Gb_12538*	687806524-687807590	651	−0.875	Nucleus	24.26	8.79
*GbWRKY28*	*Gb_12539*	688432818-688434000	759	−0.892	Nucleus	28.21	9.27
*GbWRKY29*	*Gb_05024*	327509310-327510274	702	−0.952	Nucleus	27.02	6.75
*GbWRKY30*	*Gb_05026*	328511178-328512884	1459	−0.553	Nucleus	56.47	8.60
*GbWRKY31*	*Gb_28473*	633231750-633232705	801	−0.395	Nucleus	29.58	8.52
*GbWRKY32*	*Gb_03346*	447558574-447562360	1866	−0.515	Nucleus	67.99	5.71
*GbWRKY33*	*Gb_07810*	661423975-661424813	483	−0.430	Nucleus	18.14	9.44
*GbWRKY34*	*Gb_41027*	692001348-692001991	474	−0.690	Nucleus	17.52	9.39
*GbWRKY35*	*Gb_25334*	545181619-545183218	825	−0.858	Nucleus	30.34	5.82
*GbWRKY36*	*Gb_36184*	223808232-223819523	3123	−0.260	Nucleus	111.54	8.62
*GbWRKY37*	*Gb_01391*	836924-893231	2589	−0.691	Nucleus	92.73	6.05

MW is short for Molecular weight, pI is short for Isoelectric point, GRAVY is short for Grand average of hydropathicity. GRAVY > 0 indicates that proteins were hydrophobic, while GRAVY < 0 means the proteins were hydrophilic.

**Table 2:** Ka and Ks calculation of GbWRKY gene tandem and segmental duplication.

Gene Pairs	Type of Gene Duplication	Chr. Location	Ka	Ks	Ka/Ks	Approximate Duplication Date (Mya)
GbWRKY3/GbWRKY4	Tandem duplication	Chr01	0.44224	0.58682	0.753622	0.475293
GbWRKY8/GbWRKY9	Tandem duplication	Chr01	0.399267	0.702224	0.568575	0.468139
GbWRKY23/GbWRKY24	Tandem duplication	Chr07	0.272219	0.328896	0.827675	0.286152
GbWRKY27/GbWRKY28	Tandem duplication	Chr07	0.185017	0.399258	0.463401	0.234514

## Data Availability

The datasets supporting the results of this article are included within the article.

## Supplementary Materials

The following supporting information can be downloaded at: https://www.mdpi.com/article/10.3390/genes14020343/s1, Figure S1: The phylogenetic tree of WRKY proteins from Ginkgo along with protein sequences from Arabidopsis; Figure S2: Characterization of GbWRKY genes; Figure S3: PCR amplification gels of candidate genes; Table S1: Conserved sequences of specific motifs of each subfamily; Table S2: Primer sequences of qRT-PCR; Table S3: Primer sequences for vector construction.
