# Supplementary material for: Genome-Wide Identification, Evolutionary and Functional Analyses of WRKY Family Members in Ginkgo biloba
Source: Genes (Basel). 2023 Jan 28;14(2):343. doi: 10.3390/genes14020343 (PMC9956969; doi:10.3390/genes14020343)
Supplement: Supplementary file 1 [file genes-14-00343-s001.zip › genes-2057372-supplementary.pdf]

Supplementary materials

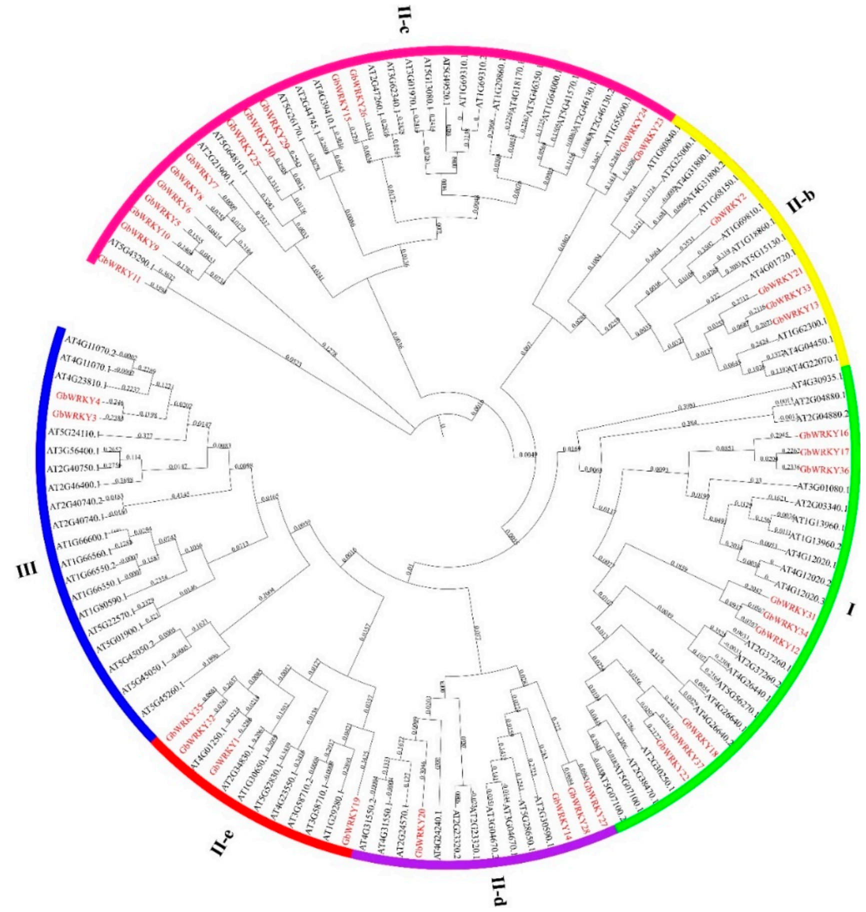

Figure S1. The phylogenetic tree of WRKY proteins from Ginkgo along with protein sequences from Arabidopsis. The tree was divided into six clades, which are marked by different colors and named as Clade I, II-b, II-c, II-d, II-e and III. The bootstrap values are indicated at each node.

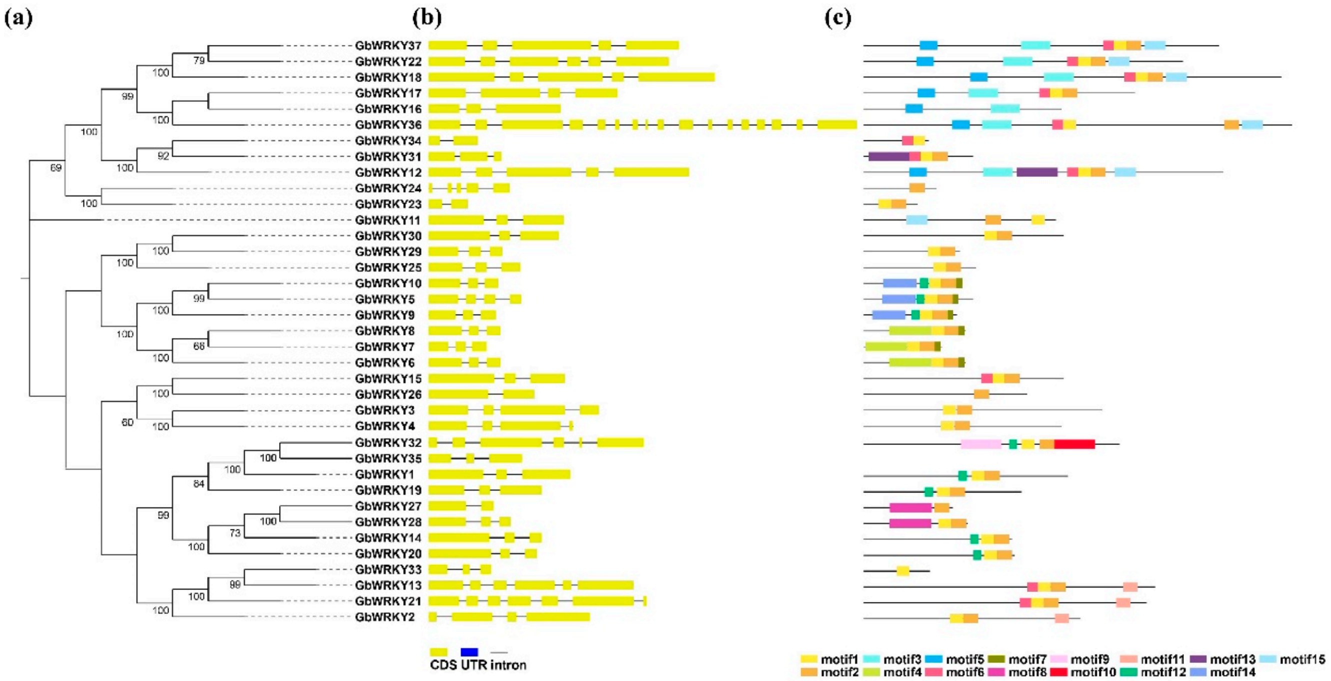

Figure S2. Characterization of GbWRKY genes. (a) Phylogenetic analysis of WRKY proteins in Ginkgo biloba. (b) Exon-intron structure of Ginkgo biloba WRKY genes. Exons are represented by yellow boxes and black lines connecting two exons represent an intron. (c) Distribution of conserved motifs identified in the 37 GbWRKY proteins. Each motif is represented by a number in a colored box. Detailed sequence information for each motif is shown in Supplementary file Table S1.

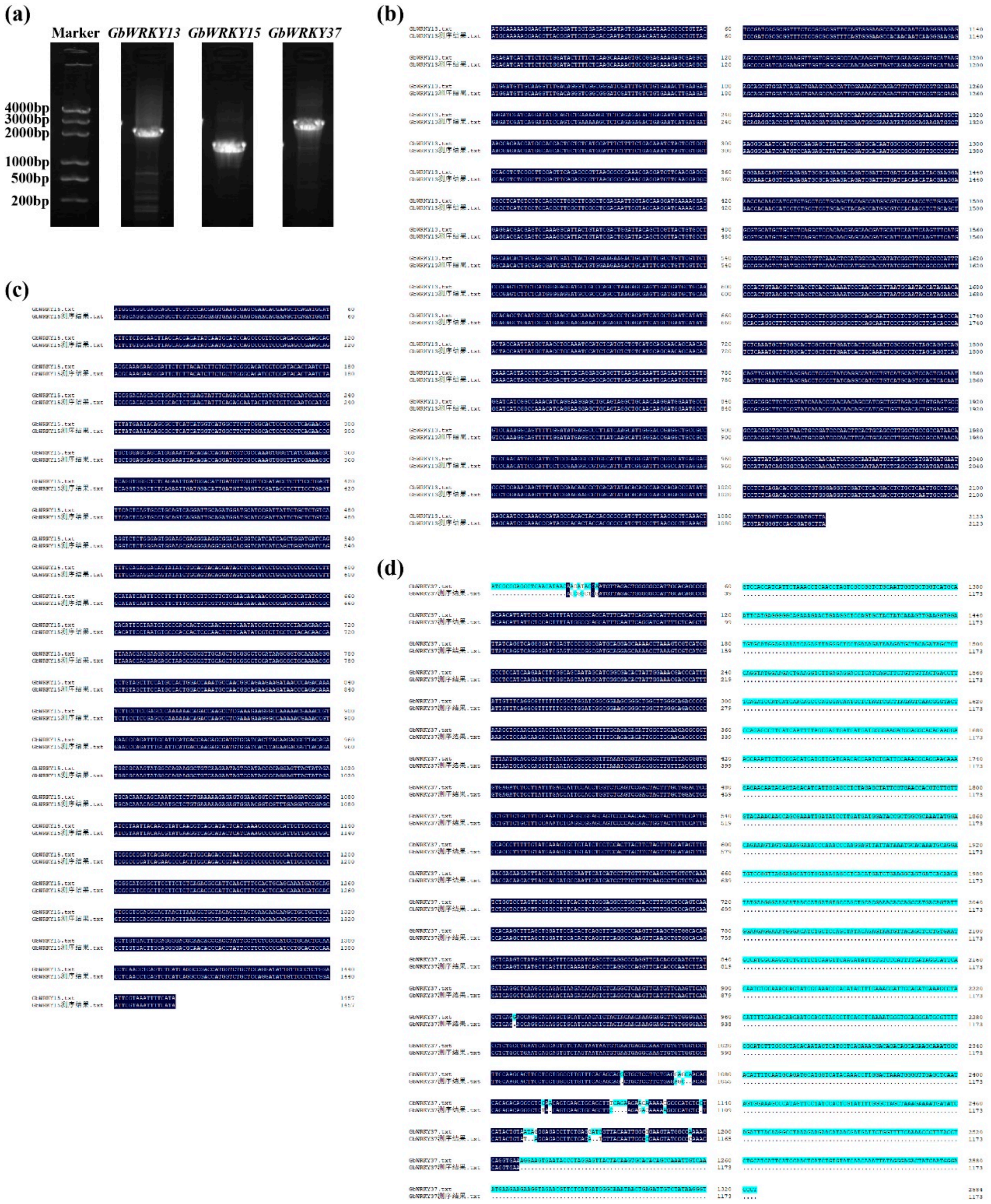

Figure S3 PCR amplification gels of candidate genes (a) and sequence comparative analysis (b-d) of candidate gene.

Table S1 Conserved sequences of specific motifs of each subfamily

**Conservative motif**	**Amino acid number**	**Conserved sequence**
1	30	DILEDGYRWRKYGQKAVKGSPHPRSYYKCT
2	36	BKGCPVRKQVERSSEDPEILITTYEGKHNHPCPAAR
3	71	VIERPSEDGYNWRKYGQKQVKGSEYPRSYYKCTHPNCPVKKKVERSHDGQI TEIVYKGVHNHPKPQPSRRM
4	100	TFSPTTDSSSKGEISLWEKSSSGQSQHLQHSRAKMPICSSSALDGDDVDQSTS GDTSDIRVLPINNRKRTIDDEENAQSKKKGTKTSTDGRKRKIIIQVR
5	41	LPIPRSPYLTIPPGLSPTTLLDSPVLLSTSQAEPSPTTGTF
6	26	KNTVEIIAAPRTIREPRVVVZTTSDV
7	14	CYVERHVYVPIPAA
8	100	NRQGHARFRKGPPPVKSHJFLDDPILHLSSGSPPLKDLDNEYMLHPDLRLRN KAEPIAQLNPLHSSSNKNKNCSHSEGEGAGAGGSKHGDFTNCHCSKRR
9	98	MFSSTVSIEKNSGENEQGVNYGFIEIEPEMHTPLNRSVEISTCEGSEDDKNSLE KKSCHTYAPLAPEFGSEVRSKLPQYSPTTSQAEAVGNTCGKEED
10	98	NSLAGSTRHNMASDKLVLEDMKSPNQDIEIGKSDNDNSVDADKNIGSDCN QLPLFISSSKAAVGQANELVPHEEEEFYEVKPNFNNQEDRQVGLRGFC
11	33	QPSLVDTVSAATAAITADPNFTAALAAAITSII
12	19	KRQCSKRRKTRQKRIIRVP
13	98	VVENAEVGAQDJQATPIVQGTQHEGKGNPSDKSYEVTISAAKEDMSTIHLV GREGLNGTILVGEQIVGHMKISFGADILPDVLSPSMSGDGREDAKDI
14	80	FDLNRFSIFSNLSSDLRPEQESSSLDTRDNHKSFYPQEFITESDHQIEAVKCSA VDGGVHMSTSEDTNGIGKGHMQKQNR
15	50	GNGPSAPAJQNNVTAPTNAIARPVSQIQDTVSHFDKHSELGNEYGKHTYM

Table S2 Primer sequences of qRT-PCR

Gene	Gene ID	Forward primer (5′-3′)	Reverse primer (3′-5′)
GbMADS1	*Gb_05176*	GCAACTGCTGAGTCTCCGTCTTC	CTGCGAGCTTGCTCATGTCTGG
GbMADS2	*Gb_02351*	GGCGGTCGATCCTCCATTCAAG	GATCGGCTGTGATAGCTGCTGTAG
GbMADS3	*Gb_00547*	GCGATATGTCCGAAGGTTCTCCAG	TGCTTGCTGCTATCCTTGTAGTCC
GbMADS4	*Gb_00545*	ATGTTGCATCCAGTGAGGAAGAGC	ATGGCATTGTTGGAGGCACAGG
GbMADS5	*Gb_40207*	TGGAGGTGTTCACATGAGCACATC	CAACACTTGTTCGCTTCGGACTTG
GbMADS6	*Gb_26411*	GGAACCAAGACCAGCACAGACG	CTCCGATGGCTTCCGTTCAACC
GbMADS7	*Gb_26412*	TGGAGACACGTCGGACATCAGAG	CGTCTGTGCTGGTCTTGGTTCC
GbMADS8	*Gb_26413*	TGGAGACACGTCGGACATCAGAG	CGTCTGTGCTGGTCTTGGTTCC
GbMADS9	*Gb_40261*	CCACAATCAGCGCAGATCATCATG	CCGCCATCTGTAACCGTCTTCC
GbMADS10	*Gb_40257*	AGGTGTTCACATGAGCACGTCTG	CCGCCATCTGTAACCGTCTTCC
GbMADS11	*Gb_17074*	GAGGAGGATGAGGAGCAGCA	AGTGCTTGCATTGCGTCAGT
GbMADS12	*Gb_36273*	CCTTCCGCTTCGTAACATCAGTCG	TCTCGTCCTCCTGGTTCTTCGC
GbMADS13	*Gb_39366*	CAGCGAACCAGACGGATATGAAGC	GCACCGCCTTCTGACTAACCTTG
GbMADS14	*Gb_16513*	GCAGCAGCAGCAGAAGTTACATTG	TTGTCATTAGCCACACTGCCATCC
GbMADS15	*Gb_01873*	GATAGCTCGCATCCTGCGTCGTC	CCTCCGGCTTGTTCTTCCAC
GbMADS16	*Gb_20926*	GTGATCAGCAAGCCGACTCC	TGCGAGAACGACCTGCAATC
GbMADS17	*Gb_31953*	CCAGTTCGTCAAGACCGACG	CATCAGAGCGGGTTGTGCAT
GbMADS18	*Gb_25118*	GATCCATCGTCAACTCCACCAAGG	TTCCATCATCGTCGTCACTTGCTG
GbMADS19	*Gb_25547*	CTTGTTCGCCGTCCTCTGTATTGG	TCCGCCTGCTTCTCCTCTGTG
GbMADS20	*Gb_01527*	GCAGCGCGGCCACAGATATG	CGGTAGGTCGAGCCACAGGAG
GbMADS21	*Gb_23334*	ACGAAGGCAATCACAACCATCCTC	TGGTGGTGGTGGTGGAAGAGC
GbMADS22	*Gb_32055*	GGCTACAACTGGCGGAAGTATGG	CAATCGACGGCTTGGCTGAGG
GbMADS23	*Gb_17623*	CACACCATCAGTGGGGATGC	CGCAGCGTTGACTTTGCTTC
GbMADS24	*Gb_15790*	AATGGTATTTCCGGCAGCAGAAGG	CCAGTTGAAGATCGGTGGCAGAG
GbMADS25	*Gb_16917*	GCAGCACAAGCAGGAGAATGATTC	CAGACTGATGCGCCTCGATAGAC
GbMADS26	*Gb_08731*	TGGCGTCGTCGAAGCTGGAG	CGGCGTGCTTGGAACTGTAGAC
GbMADS27	*Gb_12538*	TGAGCATCACCAAACCCACGATAG	CGACTCCACCTTGCGGAACTTG
GbMADS28	*Gb_12539*	CCAGGAAGCCGCTGAACAAGG	GGACTCCACCTTGGACAACGC
GbMADS29	*Gb_05024*	GTCCAAGAGCAGCAGCAACCAG	GCCGTCGTCAATGCTATCAGAGTC
GbMADS30	*Gb_05026*	AACACTCAGGAAGTTGCACACCAG	AGGCGGCTTAGACCTTGAGAGT
GbMADS31	*Gb_28473*	GAGTGGTGATGGAAGGAAGGATGC	CACGAGGTTCCCGAGACATTCTTG
GbMADS32	*Gb_03346*	CATGCCTGCAAGTAGCCGGTTC	GCTCCTCCTTAATGGCAGACAACC
GbMADS33	*Gb_07810*	TCGTCGGTTCAGGTCCAGAGATG	CAGGAGGCAGACGATGGTTGTG
GbMADS34	*Gb_41027*	GAGTGGTGATGGAAGGAAGGATGC	CACGAGGTTCCCGAGACATTCTTG
GbMADS35	*Gb_25334*	GTCGGAGGAAGTAATAGGCCAAGC	AGAACTGCTGCACCTGTAATAGCC
GbMADS36	*Gb_36184*	CACGACTCGGAACCTGCTAACAC	TGGCGGCATACACTTGAACTTGG
GbMADS37	*Gb_01391*	TCTGGTCCTAGTTCGTGCCTGTC	GCCTGAACCTGAGTGTGGAATCC

Table S3 Primer sequences for vector construction

**Gene**	**Gene ID**	**Forward primer (5′- 3′)**	**Reverse primer (3′- 5′)**
GbWRKY13	*Gb_39366*	GGGGTACCATGGAAAAAGGAAGGTTAGGGATTG	GCTCTAGATTAAGCATCGGTGGACCCATAC
GbWRKY15	*Gb_01837*	GGGGTACCGGCGAGCAGCCTCGTCCCAC	CCCTCGAGCTATGAAAATTTACGAATTCCAGAG
GbWRKY37	*Gb_01391*	GACTAGTATGGCGGAGGGTGAAGATAAC	CCCTCGAGTCAGGGTCCCATTGATAGTCT
